# Genetic Diversity of Apple Clonal Rootstocks from the Collection of the Michurinsk State Agrarian University Based on SSR Markers

**DOI:** 10.3390/plants12162991

**Published:** 2023-08-19

**Authors:** Ksenia V. Boris, Aya A. Trifonova, Maksim L. Dubrovsky, Ivan N. Shamshin, Aleksander M. Kudryavtsev

**Affiliations:** 1 Vavilov Institute of General Genetics Russian Academy of Sciences, 119333 Moscow, Russia; docboris@mail.ru (K.V.B.); element68@mail.ru (M.L.D.); ivan_shamshin@mail.ru (I.N.S.); 2456376@gmail.com (A.M.K.); 2 Michurinsk State Agrarian University, Research Center of Biotechnologies and Breeding, 393760 Michurinsk, Russia

**Keywords:** apple, clonal rootstock, microsatellite markers, genetic resources

## Abstract

The Michurinsk State Agrarian University (Michurinsk SAU) is one of the leading centers for breeding apple clonal rootstocks. A diverse collection of apple rootstocks, founded in 1930s by V.I. Budagovsky, is maintained at the Michurinsk SAU. In the present study, 87 rootstocks from this collection were analyzed using 18 SSR markers to assess their genetic diversity and relatedness. The detected polymorphism level was rather high compared to the previous estimates of apple rootstock genetic variability. A total of 199 alleles were detected with an average of 11.1 alleles per locus. Among the detected alleles, 67 (33.67%) were rare and 43 (21.61%) were unique. The average PIC value was 0.73, and the expected and observed heterozygosity averaged 0.76 and 0.69, respectively. All the studied accessions except two could be identified with the used marker set. Cluster analysis revealed several groups according to the rootstocks’ pedigrees and genetic origin. Furthermore, Structure analysis revealed two main groups of the studied rootstock accessions. No significant differentiation of the studied sample according to dwarfing ability was detected, while weak differentiation was detected according to leaf color. SSR genotyping data can be used for rootstock fingerprinting and pedigree verification and will facilitate collection management. In addition, data on the genetic diversity and structure of the studied collection may be useful for further development of the Michurinsk SAU rootstock breeding program.

## 1. Introduction

The cultivated apple (*Malus domestica* Borkh.) is the main fruit crop in many regions of the world, including Russia. The choice of a scion–rootstock combination is of great importance for the commercial production of apples. Clonal rootstock affects the growth and development of scion, resistance to biotic and abiotic stresses, productivity, and also greatly facilitates the production of high-quality planting material.

The universally recognized world standards for apple clonal rootstocks are various ‘M9’ clones. However, significant differences in climatic conditions, biological factors (e.g., diseases and pests) and management practices in different apple-growing regions of the world determine the need for new resistant and adaptive rootstocks. There are several apple rootstock breeding programs in Russia, Europe, North America, Asia, New Zealand, etc. [1,2,3,4,5,6].

Due to harsh climatic conditions (first of all extremely low winter temperatures), apple orchards in Russia for several centuries have been planted on seedling rootstocks from the most resistant local cultivars. The first apple clonal rootstock in Russia was obtained by I.V. Michurin in 1901 [7], and then this field of research was widely developed due to its significance for industrial horticulture.

The Michurinsk State Agrarian University (Michurinsk SAU) is the largest Russian center for breeding low-vigorous apple clonal rootstocks. The breeding program was organized by V.I. Budagovsky 90 years ago [8]. Its most famous clonal rootstock is ‘Paradizka Budagovskogo’ (also known as ‘Budagovsky 9’, ‘Bud. 9’ or ‘B9’), but now ’62-396’ (‘B10’) is becoming more popular. Out of 57 apple clonal rootstocks approved for cultivation in Russia, 28 were obtained here [6]. Currently, a number of new apple rootstocks selected at the Michurinsk SAU are undergoing competitive production trials.

Clonal apple rootstocks bred at the Michurinsk SAU have a unique genetic background, being complex interspecific hybrids. Various *Malus* Mill. species and their hybrids were used for breeding rootstocks as genetic sources of important characteristics: *M. baccata* (L.) Borkh., *M. prunifolia* (Willd.) Borkh., *M. sieboldii* Rehder, *M. ioensis* (Alph. Wood) Britton—resistance to main biotic and abiotic factors, including high winter hardiness and resistance to phytopatogens; *M. niedzwetzkyana* Dieck ex Koehne—anthocyanin accumulation; *M. domestica* Borkh.—lack of lateral branches and spikes of shoots in stoolbed. Thus, the Michurinsk SAU apple rootstocks collection is the largest in Russia and includes more than 300 forms.

The phenotypic identification of apple clonal rootstocks is difficult because of the limited number of available morphological traits, especially at the early stages of plant development, and because of the genetic similarity of many rootstocks. Moreover, questions of identity and parentage for some accessions are controversial due to open pollination, incorrect labeling and other problems associated with long-term collection maintenance.

Nowadays, for the efficient management of plant germplasm collections, molecular markers (e.g., SSR, SNP, DArT) are widely applied [9]. Regardless of the new genomic approaches, microsatellites (SSR (Simple Sequence Repeats) markers) based on the variability of tandemly repeated DNA sequences remain an effective and cost-efficient markers still widely used for genetic analysis [10]. To date, several hundred SSR markers have been developed in apple and other *Malus* species [11,12,13,14,15,16,17]. SSR markers have been successfully used for apple genetic resources characterization worldwide, particularly for genetic diversity assessment and genotyping [18,19,20,21,22,23,24,25,26,27].

However, despite their importance for apple production, there are few studies of rootstock genetic diversity using SSR markers. A study of 66 rootstock clones from different countries maintained at the gene bank of NIFTS, Morioka, Japan, using seven SSR markers demonstrated that these markers can be reliably used for rootstock identification, except between subclones and their original parents, the verification of parentage and the resolution of genetic relationships [28]. SSR markers were also used to study the genetic diversity and relatedness of 41 rootstocks from China, Europe and North America from Chinese germplasm collections and revealed five main groups according to genetic background and origin [29]. Still, most of the rootstocks from the Michurinsk SAU collection were not previously characterized using SSR markers.

In this study, 18 SSR markers were used to genotype 87 apple clonal rootstocks from the Michurinsk SAU collection to assess their genetic diversity and population structure. The results will help the management of the collection and the efficient use of its breeding potential.

## 2. Results

### 2.1. SSR Polymorphism and Rootstocks Identification

The 18 studied SSR loci were all polymorphic and produced one or two scorable amplicons with a total of 199 alleles. The average number of alleles per locus was 11.1, ranging from 7 (*CH05e04* and *CH03c02*) to 21 (*CH02c02a* and *CH04f10*) (Table 1). Among the detected alleles, 67 (33.67%) were rare and 43 (21.61%) were unique. No rare alleles were found at the *CH05e04* locus, while for other studied loci, the number of rare alleles ranged from 1 (*CH03c02*) to 11 (*CH04f10*). Of 87 studied accessions, 23 had unique alleles, wherein 9 unique alleles were detected for rootstock ‘G16’, 7 for ‘14-1’, and 2 for ‘M9 T337’, ‘Babarabskaya yablonya’, ‘2-14-2’, ‘2-9-94’, ‘69-6-217’ and ‘76-3-6’. Fifteen of the accessions had one unique allele each.

The observed heterozygosity (H_o_) value ranged from 0.24 (*CH03d01*) to 0.86 (*CH04f10*) and averaged 0.69. The mean expected heterozygosity (H_e_) was 0.76 and varied from 0.59 (*CH03d01*) to 0.88 (*CH04f10*). Polymorphism information content (PIC) was not lower than 0.57 (*CH03d01*) and averaged 0.73 (Table 1).

The average number of genotypes identified using one marker was 23.39, ranging from 12 (*CH01f03b*) to 46 (*CH04f10*). The 18 selected SSR markers allowed for the identification of 86 different genotypes among the 87 studied accessions. All the studied accessions had a unique SSR profile, except rootstocks ‘71-3-137’ and ‘71-3-150’ which had identical fingerprints. Moreover, three loci (*CH02c02a*, *CH02c09* and *CH04f10*) are sufficient to identify each of the 86 genotypes.

The Dice genetic similarity coefficient was calculated for each pair of studied accessions. The mean Dice coefficient value was 0.36 and ranged from 0.04 to 1. The maximum similarity was found for a pair of accessions that had the same set of alleles (‘71-3-137’ and ‘71-3-150’). For ‘B9’ and ‘57-146’, the Dice genetic similarity coefficient was also high (0.96). The lowest level of similarity was found for rootstocks ‘57-491’ and ‘86-6-12’.

### 2.2. Genetic Differentiation and Structure

**Figure 1:**
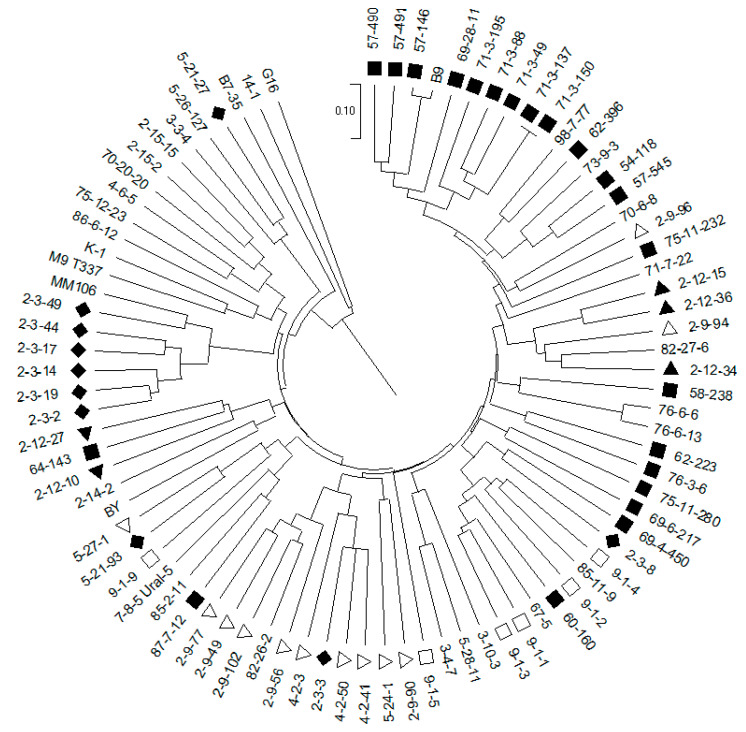
Dendrogram constructed according to SSR data using the UPGMA method and Dice coefficient for genetic similarity among 87 apple rootstock accessions. Accessions obtained from crossing: ■—‘B9’; □—‘57-157’ × ‘Stroevskoe’; ♦—‘82-27-6’; ∆—‘82-26-2’.

The UPGMA clustering method based on the Dice similarity coefficient matrix was used to elucidate genetic relationships among the studied rootstocks. The cluster analysis was able to group the accessions into several clusters (Figure 1). Accessions ‘G16’ and ‘14-1’ were grouped separately on the dendrogram, as well as the ‘B7-35’ rootstock. Accessions ‘3-3-4’, ‘5-26-127’ and ‘5-21-27’ also formed a separate cluster. The next cluster consisted of fifteen accessions and was divided into two subclusters: the first one included the ‘MM106’ rootstock and six accessions obtained from the open pollination of ‘82-27-6’. Accession ‘82-27-6’, with an unknown origin, wasn’t included in this subcluster, as well as two other forms derived from its open pollination (‘2-3-3’ and ‘2-3-8’). The second subcluster consisted of eight accessions, including ‘M9 T337’ and ‘K-1’ (‘Borovinka’ × ‘M9’), two accessions (‘2-15-15’ and ‘2-15-2’) obtained from the ‘85-8-12’ open pollination, two accessions (‘75-12-23’ and ‘86-6-12’) with unknown parentage and two accessions (‘70-20-20’ and ‘4-6-5’) with different parentage (Figure 1).

The rest of the studied rootstocks formed the fifth cluster with a complex structure. Several subclusters based mainly on rootstock pedigrees can be distinguished in this cluster. For example, subcluster consisting of ‘B9’, the 12 accessions obtained from crosses with ‘B9’ (‘57-490’, ‘57-491’, ‘57-146’, ‘69-28-11’, ‘71-3-195’, ‘71-3-88’, ‘71-3-49’, ‘71-3-137’, ‘71-3-150’, ‘62-396’, ‘54-118’ and ‘57-545’) and rootstocks ‘98-7-77’ and ‘73-9-3’ with one of the parents (‘62-396’ and ‘57-545’, respectively) were also included in this subcluster. Rootstock ‘82-26-2’ and the 9 accessions derived from ‘82-26-2’ (‘2-9-77’, ‘2-9-49’, ‘2-9-102’, ‘2-9-56’, ‘4-2-3’, ‘4-2-50’, ‘4-2-41’, ‘5-24-1’ and ‘2-9-90’) formed another subcluster together with ‘2-3-3’ (Figure 1). However, not all of the accessions obtained using ‘B9’ and ‘82-26-2’ fall into these subclusters.

**Figure 2:**
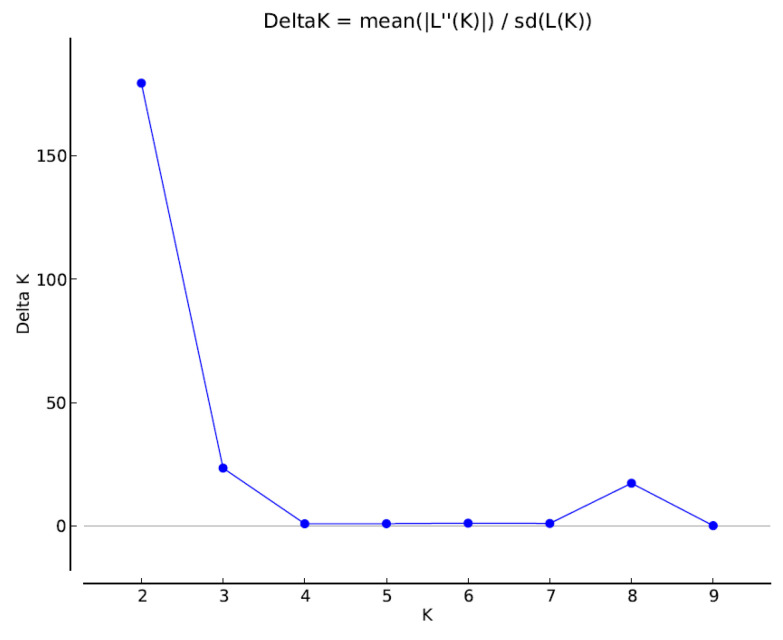
Estimation of optimal number of clusters with the deltaK method.

**Figure 3:**
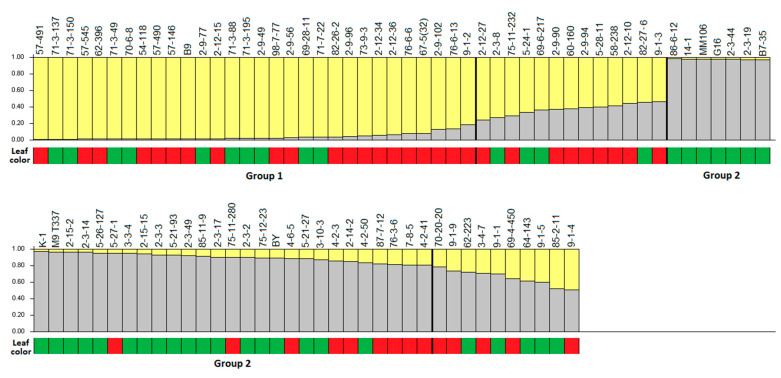
Probability of apple rootstock accession assignment to one of the groups (yellow color—group 1, gray color—group 2). Each accession is represented by a vertical bar partitioned into K = 2 segments. Under the Structure graph, the color of rootstock leaves (red or green) is shown.

The genetic structure of the 87 studied rootstock accessions was analyzed with a Bayesian clustering approach. The peak of ΔK for K = 2 corresponded to the presence of two main groups (Figure 2). The first group (yellow bars) consisted of 30 accessions originating from the Michurinsk State Agrarian University, including 12 out of 22 accessions with ‘B9’ in their parentage and ‘B9’ itself (Figure 3). Moreover, eight rootstocks (‘70-6-8’, ‘98-7-77’, ‘71-7-22’, ‘73-9-3’, ‘76-6-6’, ‘67-5(32)’, ‘76-6-13’ and ‘9-1-2’) from this group also have ‘B9’ in their pedigrees.

The second group (gray bars) consisted of 34 accessions. The composition of this group was similar to composition of the first four clusters on the dendrogram. This group included three foreign rootstocks (‘M9 T337’, ‘G16’ and ‘MM106’), two accessions (‘K-1’ and ‘B7-35’), which have ‘M9’ in their parentage, two local rootstocks obtained from wild *Malus* species (‘14-1’ and ‘Babarabskaya yablonya’). Out of ten accessions with ‘82-27-6’ in their parentage, nine were included in the second group. Other accessions from this group had different origins. The remaining 23 accessions were determined to be admixed, having components of both groups (Figure 3).

A smaller peak was detected at K = 8 (Figure 2), revealing 39 (44.8%) accessions with assigning probability to one of the groups ≥80% (Figure S1). The first group included ‘B9’ and eleven accessions with ‘B9’ in their parentage; another group (3) consisted of ‘MM106’,and six accessions from the ‘2-3’ series (‘2-3-14’, ‘2-3-17’, ‘2-3-19’, ‘2-3-44’, ‘2-3-49’ and ‘2-3-2’); another six accessions (’64-143’, ‘9-1-9’, ‘7-8-5’, ’87-7-12’, ‘5-27-1’ and ‘2-12-10’), with different pedigrees also formed a separate group (4); another group (8) included ‘9-1-1’, ‘9-1-2’, ‘9-1-3’, ‘9-1-4’ and ‘85-11-9’. Other groups consisted of only 2 and 4 accessions, and 48 accessions were admixed (Figure S1, Table S1).

The divergence between the two groups revealed by Structure analysis was evaluated by AMOVA (Table 2). This analysis revealed that a significant part of the variance (14%; *p* < 0.01) was ascribed to differences among the two detected groups.

A possible differentiation linked to phenotypic traits (leaf pigmentation and dwarfing ability) was also investigated applying AMOVA (Table 2). Only 1% (*p* < 0.09) of the total variation occurred between groups with different dwarfing abilities (VD; VD + D; D/SD + SD; SD/I + I + I/V). Furthermore, the differentiation between groups with different leaf color (red or green) accounted for about 2% (*p* < 0.01) of the total variation (Table 2).

## 3. Discussion

The studied sample of 87 apple rootstocks from the collection of the Michurinsk State Agrarian University including 3 foreign rootstocks (‘M9 T337’, ‘MM106’ and ‘G16’) and 3 rootstocks from other Russian breeding centers (‘B7-35’, ‘K-1’ and ‘7-8-5’ (‘Ural 5’)) was rather diverse: 199 alleles were detected with 11.1 alleles per locus and He = 0.76. Although there are few genetic diversity studies of apple rootstock collections, the polymorphism of the studied sample was still rather high, considering that mainly rootstocks from the Michurinsk SAU breeding program were studied.

Previously, a set of 66 rootstock clones of apples, representing random samples of rootstock varieties from major apple-growing regions in the world maintained at the apple gene bank of NIFTS, Morioka, Japan, was studied using seven SSR markers. As a result, 68 alleles were detected with an average of 9.7 alleles per locus and a mean heterozygosity value of 0.73 [28]. Another study of 41 rootstocks originating from Europe, North America and China from the Chinese germplasm centers using 62 SSR markers revealed 737 alleles with an average 11.9 alleles per locus [29].

All the 18 studied loci produced one or two discretely amplified fragments and allowed for the detection of 86 unique genotypes among 87 accessions. Rootstocks ‘71-3-137’ and ‘71-3-150’ derived from ‘58-257’ × ‘B9’ had identical alleles in all studied SSR loci. These accessions are very similar phenotypically and may be the same genotype, or the resolving power of the chosen markers is not sufficient. Still, the rest of the studied sample can be successfully identified using a set of 18 markers, even rootstocks with a common pedigree.

Marker *CH04f10* was the most polymorphic and allowed for the detection of 21 alleles and 46 genotypes (Table 1). Three loci (*CH02c02a*, *CH02c09* and *CH04f10*) were sufficient to identify each of the 86 genotypes. The obtained SSR genotyping results can be used for future data comparison between different studies.

On the whole, the informativeness of the selected marker set (average PIC value 0.73) was comparable to the studies of Japanese (average PIC value 0.81) and Chinese (average PIC value 0.606) rootstock collections [28,29]. Though, in the study by Oraguzie et al. (2005) [28], only seven markers were used, while the other study used significantly more markers (62) but the results were visualized using silver-stained polyacrylamide gels [29]. In the studies of large apple germplasm collections using different SSR marker sets, the PIC value was 0.80 for apple cultivars from different French collections and repositories [21], 0.81 for local Italian cultivars and 0.80 for local and introduced accessions from north-eastern Italy [24,25].

The genetic diversity of apple clonal rootstocks is considered limited because the Malling rootstock series was the founding germplasm for all apple rootstock breeding programs and was the source of dwarfing and precocity. The Michurinsk SAU breeding program was no exception. The first rootstocks bred at the Michurinsk SAU were obtained from ‘M8’ (East Malling, UK) and local cold hardy cultivars [8]. One of the first obtained rootstocks—‘Paradizka Budagovskogo’ (later commonly known under the name ‘B9’) (‘M8’ × ‘Krasniy Shtandart’) and ‘13-14’ (‘M8’ × ‘Tayezhnoye’)—was widely used in hybridization schemes in the 1950s–1960s.

Thus, on the dendrogram, all 22 accessions with ‘B9’ in their parentage were included in the largest cluster with a complex structure (Figure 1). Wherein 12 of them, mostly obtained in the 1950s–1970s, formed a separate subcluster. This cluster also included 18 accessions which have ‘B9’ in their pedigrees. The only exception was ‘70-20-20’ (‘57-469’ × ‘57-344’ (‘B9’ × ‘Naliv Aliy’)) which has a complex ancestry including *M. prunifolia*, *M. niedzwetzkyana* and *M. baccata*.

Out of 30 accessions in the first group in the Structure graph, 20 had ‘B9’ in their pedigrees, except ‘87-7-12’ (‘54-118’ × ‘B9’), ‘76-3-6’ (‘M27’ × ‘B9’) and ’75-11-280’ (‘B9’ open pollination), which fell into the second group (Figure 3). The foreign rootstocks and accessions derived from M series rootstocks (‘M9’, ‘M27’, ‘M4’, ‘M1’) also fell into the second Structure group as well as 8 accessions derived from ‘82-27-6’, except ‘2-3-8’ (Figure 3). Rootstock ‘82-27-6’, whose pedigree is unknown, had components of both groups. Accessions ‘G16’ (‘Ottawa 3’ × *M. floribunda*) and ‘14-1’ (*M. sieboldii* open pollination), derived from species of Section *Sorbomalus*, were also included in the second Structure group. These accessions were the most genetically differentiated from all the other studied samples (Figure 1) and had the largest number of unique alleles: nine were detected for rootstock ‘G16’ and seven for ‘14-1’.

*Malus* species are sometimes used as seedling rootstocks because of their high adaptability and tolerance to harsh environments and as sources of biotic (diseases and pests) and abiotic (frost, heat, drought, salinity) stress resistance in various apple cultivars and rootstocks breeding programs [3,4,5,8,30]. For example, *M. robusta* cv. ‘Robusta 5’ became the source of resistance to fire blight (*Erwinia amylovora* Burril), powdery mildew (*Podosphaera leucotricha* Salm.) and woolly apple aphid (*Eriosoma lanigerum* Hausm.) [31,32], and *M. sieboldii* ‘Sanashi 63’ the source of resistance to crown gall [33].

In the Michurinsk SAU breeding program, *Malus* species (*M. prunifolia*, *M. sieboldii* and *M. floribunda*) were also used as sources of resistance to heat, high soil salinity and various leaf blots. The development of rootstocks with fire blight and woolly aphid resistance is also one of the objectives of the breeding program.

Furthermore, in the Michurinsk SAU, much attention has always been paid to the high cold hardiness of the rootstock root system due to the unfavorable climatic conditions of many Russian apple-growing regions [8]. In this regard, cold resistant species and cultivars derived from the species *M. baccata* (cv. ‘Tayezhnoye’) and *M. prunifolia* (cvs. ‘Kandil Kitayka’ and ‘Pepin Shafranny’) were used as sources of high winter hardiness. As a result, most of the Michurinsk SAU rootstocks (e.g., ‘54-118’, ‘57-490’, ‘62-396’, ‘67-5(32)’, ‘70-20-20’) withstand soil temperatures down to −16 °C, which is confirmed by field observations and controlled freezing tests.

However, the use of wild *Malus* species in breeding also has disadvantages. For example, the use of *M. baccata* and American crabapples in crosses can lead to the strong lateral branching of the seedlings, which reduces the quality of the layers [34]. To avoid undesirable traits, apple cultivars are often used in breeding. The Michurinsk SAU rootstocks ‘58-238’, ‘3-3-4’, ‘3-10-3’, ‘5-21-27’, ‘5-21-93’, ‘5-24-1’, ‘5-27-1’ and family 9-1 are derived from different old, local and commercial apple cultivars (Table 3).

In addition to biotic and abiotic stress resistance, breeding efforts were directed toward developing rootstocks with high regenerative capacity, productivity and good capability for rooting layers. Dwarfing ability was also of interest for breeders, and very dwarfing rootstocks, ‘76-6-6’, ‘76-6-13’, ‘71-7-22’ and ‘9-1-1’, were developed. At the same time, many apple producers currently prefer semi-dwarf rootstocks such as ‘62-396’ (‘B10’).

A unique biological trait of Michurinsk SAU apple rootstocks is the purple-red color of young leaves and shoots, which are associated with anthocyanin accumulation. This trait is inherited from red-fleshed apple cultivars ‘Krasniy Shtandart’ (‘Red Flag’) and ‘Rubinovoe’ (‘Ruby’), derived from *M. niedzwetzkyana* by I.V. Michurin. Red leaf color is common for the Michurinsk rootstocks; however, it does not seem to provide significant adaptive advantages, though there have been studies of the effect of anthocyanins on heat resistance and tree nutrition [35,36]. Still, it is used in breeding to confirm hybridity and is convenient when removing rootstock shoots from grafted trees in a nursery.

The differentiation of accessions according to leaf colour was studied. AMOVA analysis showed weak (2%) but statistically significant (*p* < 0.01) differentiation among the groups of accessions with or without antocyanin pigmentation (Table 3). Out of 43 studied red-leaved accessions, 41 were included in the fifth cluster on the dendrogram. While the first four clusters were composed predominantly of green-leaved accessions (except ‘70-20-20’ and ‘4-6-5’) as well as the second group on the Structure graph (Figure 1 and Figure 3). The differentiation of accessions with different growth habits was also analyzed. No clear differentiation of rootstocks according to dwarfing ability was revealed, although the loci (*CH03a09* and *CH02d08*) associated with dwarfing were analyzed (Table 2).

The present study provides the first insight into the genetic variation of apple rootstocks from the collection of the Michurinsk State Agrarian University, obtained over 90 years. The studied collection is rather diverse, which is the result of the use of wild *Malus* species and hybrid forms in the breeding program along with the local and foreign old and commercial apple cultivars. SSR genotyping data provide valuable information for the proper characterization of the plant material preserved in the collection, including rootstock identification and pedigree clarification, and for the further development of the Michurinsk SAU rootstock breeding program.

## 4. Materials and Methods

### 4.1. Plant Material and DNA Extraction

The plant material for the study included 87 apple rootstocks from the collection of the Michurinsk State Agrarian University with different dwarfing abilities and leaf colors (Table 3). Total genomic DNA was extracted from fresh young leaves using Quick-DNA Plant/Seed Miniprep Kit (Zymo Research, Irvine, CA, USA), according to the manufacturer’s protocol. DNA samples extracted were quantified using a NanoDrop OneC (Thermo Scientific, Waltham, MA, USA) spectrophotometer.

### 4.2. SSR Analysis

A set of 18 SSR markers (*CH03d01, CH02c02a, CH01f02, CH01f03b, CH02c09, CH03d07, CH05e04, CHVf1, CH04e05, COL, CH01h01, CH04f10, CH01h10, CH03d08, CH03a09, CH02d08, CH02d12* and *CH03c02*) [12,14,15,37] was used for genotyping (Table 1).

PCR reactions were performed in T100 Thermal Cycler (BioRad, Hercules, CA, USA) in a final volume of 15 µL containing 20 ng of genomic DNA, 0.2 mM of each dNTP, 1.6 mM MgCl_2_, 1×PCR buffer, 0.3 µM forward and reverse primers and 0.5 U of BioTaq DNA polymerase (Dialat Ltd., Moscow, Russia). Forward primers were labeled with four different fluorescent dyes (6-FAM, R6G, TAMRA and ROX). All the SSR loci were amplified as described in Gianfranceschi et al. [12] and Liebhard et al. [14] with minor modifications. All microsatellites were amplified separately and combined in multiplexes depending on the size range after PCR products were checked on 1.5% agarose gels in 1X TBE buffer and visualized by staining with ethidium bromide to test for the presence of PCR products.

Fluorescently labeled PCR products were separated by capillary electrophoresis on ABI Prism 3500 (Applied Biosystems, Waltham, MA, USA). Fragment sizes were determined using GeneMapper v4.0 software (Applied Biosystems, MA, USA).

### 4.3. Data Analysis

The frequencies of observed microsatellite alleles (rare—less than 5% of the accessions; and unique—less than 1%) and the expected (H_e_) and observed (H_o_) heterozygosity value of each microsatellite were measured using the GENALEX 6.41 software [38].

Based on the frequencies of observed microsatellite alleles, the polymorphism information content (PIC) was calculated as:

$$PIC=1-\sum_{i=1}^{l}P_{i}^{2}-\sum_{i=1}^{l-1}\sum_{j=i+1}^{l}2P_{i}^{2}P_{j}^{2}$$

where *P_i_* and *P_j_* are the population frequency of the *i*th and *j*th allele [39] in MS Excel.

Dice coefficients were used for genetic similarity estimation and to visualize genetic relationships among the studied accessions by an UPGMA (unweighted pair group method with arithmetic mean) clustering method, using MEGA 11 [40].

Genetic structure analysis of the collection was performed using Structure v.2.3.4 software [41]. From 1 to 10 clusters (K) with 5 replicates for each K were tested. The number of possible clusters was found as a result of 1,000,000 iterations of the Markov chain Monte Carlo, taking into account genetic admixture and correlated allele frequencies. The first 300,000 generations were eliminated (burn-in). The optimal number of clusters was determined as recommended by Evanno et al. [42] using the online program Structure Harvester [43]. The genotypes were assigned to one of the groups when the assigning probability was ≥80%.

The divergence between the groups revealed by Structure analysis and differentiation of accessions depending on leaf color (red/green) and dwarfing ability (VD; VD/D + D; D/SD + SD; SD/I + I + I/V) was investigated with Analysis of Molecular Variance (AMOVA) in the GENALEX 6.41 software [38]. The threshold for statistical significance was determined by running 999 permutations.

## Figures and Tables

**Table 1:** Genetic diversity parameters calculated for 18 SSR markers in 87 apple rootstock accessions.

Locus	Number of Alleles	Allele Size Range, (bp)	H_o_	H_e_	PIC	Rare Alleles	Unique Alleles	Number of Genotypes
*CH03d01*	11	89–110	0.24	0.59	0.57	4	3	18
*CH02c02a*	21	130–247	0.76	0.87	0.86	8	7	41
*CH01f02*	12	172–207	0.74	0.70	0.67	6	3	24
*CH01f03b*	8	136–176	0.77	0.71	0.66	3	1	12
*CH02c09*	8	233–257	0.44	0.77	0.73	2	1	18
*CH03d07*	11	170–229	0.83	0.82	0.79	3	3	26
*CH05e04*	7	134–258	0.84	0.81	0.78	0	1	18
*CHVf1*	10	132–171	0.82	0.85	0.83	3	0	32
*CH04e05*	14	176–229	0.85	0.83	0.81	4	4	32
*COL*	8	215–243	0.26	0.66	0.60	2	2	14
*CH01h01*	10	104–130	0.64	0.78	0.75	4	2	20
*CH* *04f10*	21	175–317	0.86	0.88	0.87	11	4	46
*CH* *01h10*	9	92–118	0.77	0.66	0.62	3	3	14
*CH03d08*	10	129–175	0.84	0.86	0.85	2	0	31
*CH03a09*	10	121–143	0.82	0.75	0.72	2	2	22
*CH02d08*	12	215–259	0.67	0.65	0.61	6	3	21
*CH02d12*	10	174–202	0.82	0.77	0.73	3	3	18
*CH03c02*	7	106–134	0.44	0.70	0.65	1	1	14
All	199	-	-	-	-	67	43	-
Average	11.1	-	0.69	0.76	0.73	3.72	2.39	23.39

**Table 2:** Analysis of molecular variance (AMOVA) based on 18 SSR loci of 87 studied rootstock accessions among inferred groups.

Source of Variation	d.f.	Sum of Squares	Estimated Variability	Percentage of Variation	*p* (Rand ≥ Data)
Structure group based					
Among groups	1	89.24	2.36	14	0.01
Within groups	62	865.39	13.96	86	0.01
Total	63	954.63	16.32	100	0.01
Leaf pigmentation based					
Among groups	1	29.33	0.33	2	0.01
Within groups	85	1266.49	14.90	98	0.01
Total	86	1295.82	15.23	100	0.01
Dwarfing ability based					
Among groups	3	50.77	0.10	1	0.09
Within groups	83	1245.04	15.00	99	0.09
Total	86	1295.82	15.10	100	0.09

**Table 3:** Apple rootstocks taken into analysis.

№	Accession	Parentage	Leaf Pigmentation *	Dwarfing Ability **
1	Budagovsky 9 (Paradizka Budagovskogo, PB, B9, Bud9)	M8 × Krasniy Shtandart (Red Flag)	+	D
2	54-118	B9 × 13-14	+	I
3	57-146	B9 open pollination	+	D
4	57-490	B9 × 13-14	+	I/V
5	57-491	B9 × 13-14	+	D
6	57-545	B9 × 13-14	+	I
7	58-238	B9 × Naliv Aliy	+	I
8	60-160	B9 × 49-290	+	D/SD
9	62-223	Anoka × B9	–	I
10	62-396 (B10)	13-14 × B9	+	SD
11	64-143	B9 × 57-290	–	I
12	67-5(32)	54-83 open pollination	+	SD/I
13	69-4-450	B9 × *M. niedzwetzkyana*	+	D/SD
14	69-6-217	B9 × Kitayka Rozovaya	–	SD
15	69-28-11	58-257 × B9	–	I
16	70-6-8	54-83 × 57-344	–	I
17	70-20-20	57-469 × 57-344	+	I/V
18	71-3-49	58-257 × B9	–	I
19	71-3-88	58-257 × B9	–	I
20	71-3-137	58-257 × B9	–	I
21	71-3-150	58-257 × B9	–	I
22	71-3-195	58-257 × B9	–	I
23	71-7-22	57-531 × 57-233	–	VD
24	73-9-3	57-545 × 57-366	+	SD
25	75-11-232	B9 open pollination	+	SD
26	75-11-280	B9 open pollination	+	D
27	75-12-23	A2 open pollination	–	D
28	76-3-6	M27 × B9	+	VD/D
29	Malysh Budagovskogo (MB, 76-6-6)	57-344 × 57-490	+	VD
30	76-6-13	57-344 × 57-490	+	VD
31	82-26-2	-	+	SD/I
32	82-27-6	-	–	SD/I
33	85-2-11	3-4-98 × 54-118	–	I
34	85-11-9	70-5-10 × 54-118	–	D
35	86-6-12	-	–	I
36	87-7-12	54-118 × B9	+	I
37	98-7-77	62-396 × 58-199	+	I
38	2-3-2	82-27-6 open pollination	–	SD
39	2-3-3	82-27-6 open pollination	–	SD
40	2-3-8	82-27-6 open pollination	–	D
41	2-3-14	82-27-6 open pollination	–	D
42	2-3-17	82-27-6 open pollination	–	I
43	2-3-19	82-27-6 open pollination	–	I
44	2-3-44	82-27-6 open pollination	–	SD/I
45	2-3-49	82-27-6 open pollination	–	SD
46	2-9-49	82-26-2 open pollination	–	SD
47	2-9-56	82-26-2 open pollination	+	D/SD
48	2-9-77	82-26-2 open pollination	–	SD
49	2-9-90	82-26-2 open pollination	+	D
50	2-9-94	82-26-2 open pollination	+	SD
51	2-9-96	82-26-2 open pollination	+	SD
52	2-9-102	82-26-2 open pollination	+	I
53	2-12-10	82-11-5 open pollination	+	I
54	2-12-15	82-11-5 open pollination	+	I
55	2-12-27	82-11-5 open pollination	+	SD/I
56	2-12-34	82-11-5 open pollination	+	D/SD
57	2-12-36	82-11-5 open pollination	+	D/SD
58	2-14-2	82-26-52 × 60-160	+	VD/D
59	2-15-2	85-8-12 open pollination	–	SD/I
60	2-15-15	85-8-12 open pollination	–	SD/I
61	3-3-4	85-6-5 × Spartan	–	I
62	3-4-7	62-396 open pollination	+	SD
63	3-10-3	82-11-2 × Wealthy	–	I
64	4-2-3	82-52-6 × 82-26-2	+	I
65	4-2-41	82-52-6 × 82-26-2	+	D
66	4-2-50	82-52-6 × 82-26-2	–	I
67	4-6-5	83-11-10 open pollination	+	SD/I
68	5-21-27	82-27-6 × Zhigulevskoe	–	I
69	5-21-93	82-27-6 × Zhigulevskoe	–	I
70	5-24-1	82-26-2 × Orlik	–	I
71	5-26-127	-	–	D
72	5-27-1	Zhigulevskoe × 82-26-2	+	I
73	5-28-11	82-57-8 × *M. baccata*	+	SD
74	9-1-1	57-157 × Stroevskoe	–	VD
75	9-1-2	57-157 × Stroevskoe	+	D/SD
76	9-1-3	57-157 × Stroevskoe	+	D/SD
77	9-1-4	57-157 × Stroevskoe	+	SD
78	9-1-5	57-157 × Stroevskoe	–	SD
79	9-1-9	57-157 × Stroevskoe	+	SD
80	14-1	*M. sieboldii* open pollination	–	I
81	Babarabskaya yablonya (BY)	*M. turkmenorum*	–	I
82	M9 T337	M9 clone	–	D
83	MM106	M1 × Northern Spy	–	I
84	G16	Ottawa 3 × M. floribunda	–	D
85	K-1	Borovinka × M9	–	SD
86	B7-35	M4 × M9	–	D/SD
87	7-8-5 (Ural-5)	57-469 open pollination	+	I

* «+»—red color (antocyanin pigmentation); «–»—green color. ** VD—very dwarfing; D—dwarfing; SD—semi-dwarfing; I—intermediate; V—vigorous.

## Data Availability

Raw data are available on request from the corresponding author.

## Supplementary Materials

The following supporting information can be downloaded at https://www.mdpi.com/article/10.3390/plants12162991/s1: Figure S1: Probability of apple rootstock accession assignment to one of the groups. Each accession is represented by a vertical bar partitioned into K = 8 segments. Table S1: Assignment of the studied apple rootstock accessions to the cluster on the dendrogram and to the group (K = 2 and K = 8) defined by Structure (assignment probability ≥80%). The color of the cells corresponds to the color of the Structure group on the graphs (Figure 3 and Figure S1).
