# Supplementary material for: Genetic Diversity of Apple Clonal Rootstocks from the Collection of the Michurinsk State Agrarian University Based on SSR Markers
Source: Plants (Basel). 2023 Aug 19;12(16):2991. doi: 10.3390/plants12162991 (PMC10458785; doi:10.3390/plants12162991)
Supplement: Supplementary file 1 [file plants-12-02991-s001.zip › plants-2500658-supplementary.pdf]

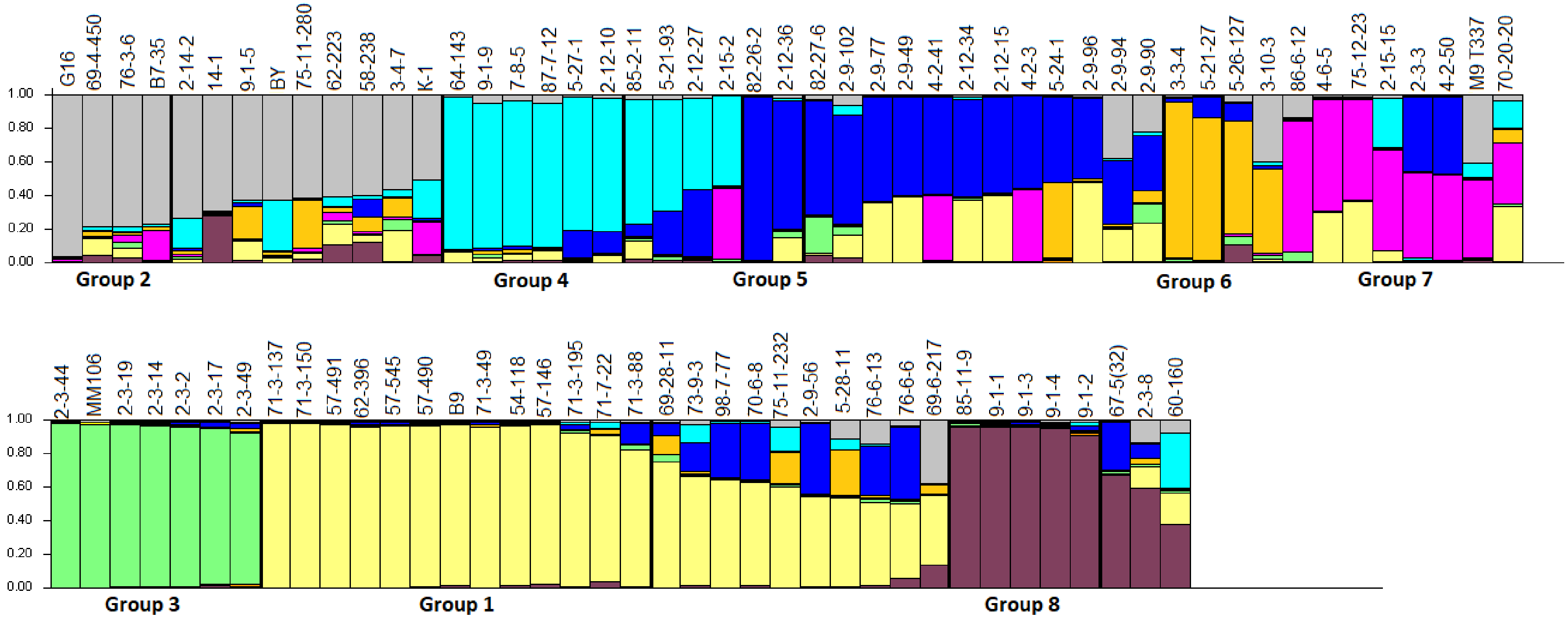

Figure S1. Probability of apple rootstock accession assignment to one of the groups. Each accession is represented by a vertical bar partitioned into K = 8 segments.

Table S1. Assignment of the studied apple rootstock accessions to the cluster on the dendrogram and to the group (K = 2 and K = 8) defined by Structure (assignment probability ≥80%). The color of the cells corresponds to the color of the Structure group on the graphs (Figure 3, Figure S1).

№	Accession	Parentage	Cluster/ Subcluster	*k*=2	*k*=8
1	Budagovsky 9 (Paradizka Budagovskogo, PB, B9, Bud9)	M8 × Krasniy Shtandart (Red Flag)	5	1	1
2	54-118	B9 × 13-14	5	1	1
3	57-146	B9 open pollination	5	1	1
4	57-490	B9 × 13-14	5	1	1
5	57-491	B9 × 13-14	5	1	1
6	57-545	B9 × 13-14	5	1	1
7	58-238	B9 × Naliv Aliy	5	admx	admx
8	60-160	B9 × 49-290	5	admx	admx
9	62-223	Anoka × B9	5	admx	admx
10	62-396 (B10)	13-14 × B9	5	1	1
11	64-143	B9 × 57-290	5	admx	4
12	67-5(32)	54-83 open pollination	5	1	admx
13	69-4-450	B9 × *M. niedzwetzkyana*	5	admx	2
14	69-6-217	B9 × Kitayka Rozovaya	5	admx	admx
15	69-28-11	58-257 × B9	5	1	admx
16	70-6-8	54-83 × 57-344	5	1	admx
17	70-20-20	57-469 × 57-344	4/1	admx	admx
18	71-3-49	58-257 × B9	5	1	1
19	71-3-88	58-257 × B9	5	1	1
20	71-3-137	58-257 × B9	5	1	1
21	71-3-150	58-257 × B9	5	1	1
22	71-3-195	58-257 × B9	5	1	1
23	71-7-22	57-531 × 57-233	5	1	1
24	73-9-3	57-545 × 57-366	5	1	admx
25	75-11-232	B9 open pollination	5	admx	admx
26	75-11-280	B9 open pollination	5	2	admx
27	75-12-23	A2 open pollination	4/1	2	admx
28	76-3-6	M27 × B9	5	2	2
29	Malysh Budagovskogo (MB, 76-6-6)	57-344 × 57-490	5	1	admx
30	76-6-13	57-344 × 57-490	5	1	admx
31	82-26-2	-	5	1	5
32	82-27-6	-	5	admx	admx
33	85-2-11	3-4-98 × 54-118	5	admx	admx
34	85-11-9	70-5-10 × 54-118	5	2	8
35	86-6-12	-	4/1	2	admx
36	87-7-12	54-118 × B9	5	2	4
37	98-7-77	62-396 × 58-199	5	1	admx
38	2-3-2	82-27-6 open pollination	4/2	2	3
39	2-3-3	82-27-6 open pollination	5	2	admx
40	2-3-8	82-27-6 open pollination	5	admx	admx
41	2-3-14	82-27-6 open pollination	4/2	2	3
42	2-3-17	82-27-6 open pollination	4/2	2	3
43	2-3-19	82-27-6 open pollination	4/2	2	3
44	2-3-44	82-27-6 open pollination	4/2	2	3

45	2-3-49	82-27-6 open pollination	4/2	2	3
46	2-9-49	82-26-2 open pollination	5	1	admx
47	2-9-56	82-26-2 open pollination	5	1	admx
48	2-9-77	82-26-2 open pollination	5	1	admx
49	2-9-90	82-26-2 open pollination	5	admx	admx
50	2-9-94	82-26-2 open pollination	5	admx	admx
51	2-9-96	82-26-2 open pollination	5	1	admx
52	2-9-102	82-26-2 open pollination	5	1	admx
53	2-12-10	82-11-5 open pollination	5	admx	4
54	2-12-15	82-11-5 open pollination	5	1	admx
55	2-12-27	82-11-5 open pollination	5	admx	admx
56	2-12-34	82-11-5 open pollination	5	1	admx
57	2-12-36	82-11-5 open pollination	5	1	5
58	2-14-2	82-26-52 × 60-160	5	2	admx
59	2-15-2	85-8-12 open pollination	4/1	2	admx
60	2-15-15	85-8-12 open pollination	4/1	2	admx
61	3-3-4	85-6-5 × Spartan	3	2	6
62	3-4-7	62-396 open pollination	5	admx	admx
63	3-10-3	82-11-2 × Wealthy	5	2	admx
64	4-2-3	82-52-6 × 82-26-2	5	2	admx
65	4-2-41	82-52-6 × 82-26-2	5	2	admx
66	4-2-50	82-52-6 × 82-26-2	5	2	admx
67	4-6-5	83-11-10 open pollination	4/1	2	admx
68	5-21-27	82-27-6 × Zhigulevskoe	3	2	6
69	5-21-93	82-27-6 × Zhigulevskoe	5	2	admx
70	5-24-1	82-26-2 × Orlik	5	admx	admx
71	5-26-127	-	3	2	admx
72	5-27-1	Zhigulevskoe × 82-26-2	5	2	4
73	5-28-11	82-57-8 × *M. baccata*	5	admx	admx
74	9-1-1	57-157 × Stroevskoe	5	admx	8
75	9-1-2	57-157 × Stroevskoe	5	1	8
76	9-1-3	57-157 × Stroevskoe	5	admx	8
77	9-1-4	57-157 × Stroevskoe	5	admx	8
78	9-1-5	57-157 × Stroevskoe	5	admx	admx
79	9-1-9	57-157 × Stroevskoe	5	admx	4
80	14-1	*M. sieboldii* open pollination	1	2	admx
81	Babarabskaya yablonya (BY)	*M. turkmenorum*	5	2	admx
82	M9 T337	M9 clone	4/1	2	admx
83	MM106	M1 × Northern Spy	4/2	2	3
84	G16	Ottawa 3 × *M. floribunda*	1	2	2
85	K-1	Borovinka × M9	4/1	2	admx
86	B7-35	M4 × M9	2	2	2
87	7-8-5 (Ural-5)	57-469 open pollination	5	2	4
